# Effect analysis of repeated transcranial magnetic stimulation combined with fluoxetine in the treatment of first-episode adolescent depression

**DOI:** 10.3389/fpsyt.2024.1397706

**Published:** 2024-06-13

**Authors:** Long Jiao, Tingting Chen, Yuanyuan Huang, Xiaoqin Huang

**Affiliations:** ^1^ Department of Psychology, The First Affiliated Hospital of Anhui Medical University, Heifei, China; ^2^ Department of Child Psychology, Anhui Provincial Children’s Hospital, Heifei, China; ^3^ Laboratory of Medical Test, Hefei Technology College, Heifei, China

**Keywords:** HAMD-17, CGI-I, repeated transcranial magnetic stimulation, fluoxetine, WCST

## Abstract

**Objectives:**

This study aims to evaluate the efficacy of repeated transcranial magnetic stimulation (rTMS) combined with fluoxetine in enhancing the early antidepressant response in first-episode adolescent depression cases, providing insights for patient diagnosis and treatment.

**Methods:**

One hundred and thirty-five adolescents experiencing their first depressive episode were randomly assigned to either a sham group treated with fluoxetine or to low or high repetitive transcranial magnetic stimulation (rTMS) groups receiving both rTMS and fluoxetine. Therapeutic effects were assessed by comparing changes in Hamilton Depression Scale (HAMD-17) scores, cognitive function scores from the Wisconsin Card Sorting Test (WCST), and Clinical Global Impression-improvement (CGI-I) scores, along with recording adverse reactions.

**Results:**

The total effectiveness rate in the rTMS groups (Low, 95.56%; High, 97.78%) was significantly higher than in the Sham rTMS group (80%) (F = 11.15, P<0.0001). Post-treatment, not only the Low but also the High rTMS group exhibited more significant reductions in HAMD-17 (Low, 21.05; High, 21.45) and CGI-I scores (Low, 3.44; High, 3.60) compared to the Sham rTMS group (HAMD-17, 16.05; CGI-I, 2.57) (two weeks: F = 7.889, P = 0.0006; four weeks: F = 15.900, P<0.0001). Additionally, the two rTMS groups exhibited fewer erroneous responses and persistent errors in the WCST and completed more WCST categorizations than the Sham rTMS group. There was no significant difference in adverse reaction rates between the groups (F=4.421, P=0.0794).

**Conclusions:**

The combination of fluoxetine with rTMS demonstrates enhanced therapeutic effectiveness in treating adolescent depression, effectively controlling disease progression, reducing depressive symptoms, and improving cognitive function, making it a valuable clinical approach.

## 1 Introduction

Depression is a widespread psychological disorder characterized by persistent low mood, sluggish cognition, and diminished volitional activity. Patients often experience cognitive impairment and somatic symptoms (1, 2). Epidemiological studies indicate that the incidence of initial-onset depression in adolescents is approximately 8%, with this rate increasing annually, posing a significant risk to the physical and mental health of this young demographic (3–5). Adolescent depression is marked by complex clinical presentations, protracted durations, and frequent recurrences (6, 7). Untreated or improperly managed, it can evolve into a chronic and refractory condition. The etiology of depression is multifaceted and may differ between adolescents and adults. For adolescents, depression may stem from genetic factors, psychosocial influences, and a history of mental illness, while in adults, it is often triggered by stressful life events, pessimistic personality traits, chronic diseases, and substance abuse. The symptoms vary; adolescents primarily exhibit symptoms of persistent sadness, dysphoria, and a lack of motivation and interest in learning. Some adolescents may experience frequent tantrums, are easily irritable and provoked, and display relatively strong emotional outbursts. In contrast, adult depression is primarily characterized by significant and lasting emotional depression and pessimism. The response to treatment also differs; adolescents generally have a better prognosis following active treatment, while adults, if not engaging actively in treatment, may experience worsening conditions. In severe cases, this can lead to self-harm, suicidal tendencies, or other serious health impacts. Adolescent depression is distinct from adult depression in its causes, symptoms, and response to treatment, necessitating early, proactive, and effective intervention to enhance clinical recovery rates and significantly improve patient outcomes (8, 9).

Currently, pharmacological and psychological therapies are the primary treatments for initial-onset adolescent depression, with a focus on pharmacological interventions. In clinical settings, serotonin reuptake inhibitors (SSRIs), such as fluoxetine, are widely used. fluoxetine is noted for its superior bioavailability and rapid absorption, exhibiting robust clinical effectiveness and safety (10). It selectively targets the serotonin (5-HT) transporter, effectively blocking 5-HT reuptake and enhancing serotonin transmission in the brain, thus yielding rapid antidepressant effects (11). However, like other antidepressants, fluoxetine may experience challenges such as delayed onset of action, poor adherence, and non-response in some patients.

Repetitive Transcranial Magnetic Stimulation (rTMS) is recognized in various guidelines as providing Level 1 efficacy evidence for treating adult depression and is approved by the U.S. Food and Drug Administration (FDA) for this purpose, with an effectiveness rate of approximately 50% (12–15). Research into rTMS for adolescent depression, anxiety disorders, attention deficit hyperactivity disorder, and autism spectrum disorders is limited (16). Although not FDA-approved for adolescent depression, rTMS serves as an effective alternative for adolescents who show minimal response to pharmacological treatments. rTMS primarily affects the prefrontal cortex, notably the dorsolateral prefrontal cortex (DLPFC), which is crucial in emotional regulation and cognitive functions and often exhibits functional anomalies in depression patients. rTMS applies magnetic pulses of specific intensity and frequency directly to the cerebral cortex, targeting the DLPFC, modifying activity in the targeted brain regions, promoting neurotransmitter production, and inducing neuroplastic effects (17–19). Depending on the stimulation frequency, rTMS is categorized into high frequency (typically above 1Hz) and low frequency (at or below 1Hz). High-frequency rTMS increases neural activity in the targeted left DLPFC, whereas low-frequency rTMS treduces it in the right DLPFC (20–22). By targeting the DLPFC, rTMS can adjust neural networks in the brain, thus enhancing mood and cognitive functions. Existing studies affirm that rTMS yields beneficial outcomes in treating adult depression, motivating further research into its efficacy for adolescent depression (23, 24). Thus, this study aims to provide valuable insights for adolescents suffering from depression by utilizing a combination of rTMS and fluoxetine as a more effective treatment strategy and to support clinical treatment.

## 2 Material and methods

### 2.1 Participant details

One hundred and thirty-five first-episode adolescent depression patients, admitted to our hospital from June 2022 to April 2024, were selected as participants and randomly assigned to a Sham rTMS group, a low rTMS group, or a high rTMS group, with 45 individuals in each. The Sham rTMS group included 18 males and 27 females, averaging 14.91±1.55 years in age, with a range from 12 to 18 years. The average duration of illness was 6.16±1.76 months, with durations spanning 3 to 12 months. The low rTMS group comprised 20 males and 25 females, with an average age of 15.07±1.39 years and disease durations ranging from 3 to 12 months, averaging 5.73±1.70 months. The high rTMS group consisted of 28 males and 17 females, with an average age of 14.87 ± 1.31 years and illness durations also between 3 and 12 months, averaging 5.23 ± 1.55 months (**Table 1**). No significant demographic differences were noted between the groups (Age, p=0.7271; Sex, p=0.8884, Education, p=0.6826), ensuring comparability within the study.

**Table 1:** Demographic data of the participants in the present study.

Variables	Sham rTMS group (n=45)	Low rTMS group (n=45)	High rTMS group (n=45)	P value
	Mean (standard deviation)			
**Age (year)**	14.91 (1.55)	15.07 (1.39)	14.80 (1.80)	0.7271
**Sex (n, male/female)**	18/27	20/25	20/25	0.8884
**Education (year)**	7.80 (1.50)	7.93 (1.42)	7.64 (1.76)	0.6826

Inclusion criteria: ① Diagnosis according to the International Classification of Diseases (10th edition) criteria for adolescent depression (F32.900); ② A score of ≥17 on the 17-item Hamilton Depression Scale (HAMD-17); ③ Age between 12–18 years; ④ No prior use of antipsychotic drugs or antidepressants; ⑤ Guardian informed consent.

Exclusion criteria: (1) Presence of severe physical illness; (2) Presence of psychotic symptoms; (3) Previous head injury; (4) Intracranial metal foreign body; (5) History of epilepsy or family history of epilepsy; (6) Pacemakers, stents, and cochlear implants; (7) History of drug or alcohol dependence; (8) Previous repetitive transcranial magnetic stimulation therapy; (9) Inability to cooperate.

### 2.2 Drug treatment

All patients received fluoxetine (20mg×14 capsules, approval number: H19980114, Shanghai Zhongxi Pharmaceutical Co., LTD.). The initial daily dosage of 20 mg was increased to 40 mg after two weeks to achieve effective control of mental symptoms without obvious side effects. This regimen lasted for four weeks.

### 2.3 rTMS protocol

The rTMS treatment was performed using a Magneuro 60 magnetic stimulator (VISHEE Inc., Nanjing, China) equipped with the figure-of-eight coil device. The exact location of the stimulation target was determined by jointly integrating an international 10–10 system and individualized 3D structural MRI. Any self-reported adverse event was recorded after each session of treatment. Alongside the medication regimen, patients underwent repetitive transcranial magnetic stimulation therapy. Preparatory steps involved seating the patient, removing any metallic objects, ensuring the patient wore headphones to minimize disturbances, and controlling indoor lighting. Patients were instructed to keep their heads still during the procedure.

Sham rTMS group: Alongside the medication regimen, the Sham rTMS group was combined with pseudo-stimulation therapy, and the pseudo-magnetic stimulation coil with the same appearance shape as the Low rTMS group was used (uniformly equipped by the instrument manufacturer), which could simulate the sound and rhythm of transcranial magnetic stimulation, but did not generate magnetic field. The parameters (intensity and stimulation settings) were identical to those used in the Low rTMS group.

Low rTMS group: Alongside the medication regimen, stimulation targeted the right dorsolateral prefrontal cortex (DLPFC). The stimulation scheme was as follows: 1 Hz frequency; 80% of the resting motor threshold (RMT); 20-second intermittent periods, 60 series, totaling 1200 pulses. Each session lasted 20 minutes, conducted five days a week with a 2-day break, over four weeks.

High rTMS group: The high rTMS group will receive high-intensity rTMS in addition to the medication regimen and the targeted position is the left dorsolateral prefrontal cortex (DLPFC). The High rTMS group will use the same equipment as the Low rTMS group for intervention, but the treatment parameters of the frequency are 10 Hz, stimulation intensity is 110% RMT, pulse number, interval number, and conducted method are consistent with the Low rTMS group.

### 2.4 Ethical approval

This research was approved by the ethical committee of Anhui Provincial Children’s Hospital. All potential participants were given detailed descriptive information about the study and were informed about the voluntary and confidential nature of their participation. All guardians provided written informed consent.

### 2.5 Clinical assessments

Clinical efficacy and adverse reactions were evaluated before treatment, as well as two and four weeks post-treatment.

Mental symptoms: The Hamilton Depression Scale (HAMD-17) was utilized to assess patients’ depressive symptoms. HAMD-17 scale scores greater than or equal to 17 can be diagnosed as depression, the higher the score, the higher the degree of depression.

Treatment Efficacy Rate: A reduction of ≥ 75% in HAMD-17 scores was classified as recovery. A decrease of 50–74% was considered significantly effective, and a 25–49% reduction was deemed effective. A reduction rate of < 25% was categorized as ineffective. The total response rate was calculated as (recovered + significantly effective + effective)/total cases ×100%.

Overall Clinical Efficacy: The Clinical Global Impression-improvement (CGI-I) was employed to evaluate the therapeutic effect, with lower scores indicating better clinical outcomes.

Cognitive Function: Cognitive performance was assessed using the Wisconsin Card Sorting Test (WCST) (25–27), which included metrics such as total tests administered, number of correct responses, number of random errors, number of persistent errors, and completion of classification tasks.

Adverse reactions: Adverse reactions were evaluated using the Treatment-Emergent Symptom Scale (TESS).

### 2.6 Statistical analysis

Data analysis was conducted using SPSS 24.0 software (IBM Corp., Armonk, NY, USA). The paired T-test was employed for age, sex, and education comparisons and WCST scores before and after treatment. The total clinical efficacy, HAMD-17 scores, CGI-I scores, and adverse reactions were compared among the three groups using one-way analysis of variance (ANOVA). Count data were represented as cases (%) and compared using the X^2^ test. A P-value < 0.05 was considered statistically significant.

## 3 Result

### 3.1 Comparison of total clinical efficacy between three groups

The total effective rate of one-way ANOVA showed that the Low and High rTMS group’s effective rate was 95.56% and 97.78%, respectively, significantly higher than the Sham rTMS group’s 80.00% (F = 11.15, P <0.0001), as illustrated in **Table 2**.

**Table 2:** Comparison of total clinical efficacy between three groups [n=135, n (%)].

Group	Cases	Significantly effective	Effective	Ineffective	Total effective rate
**Sham rTMS group**	45	23	13	9	36 (80.00%)
**Low rTMS group**	45	30	13	2	43 (95.56%)
**High rTMS group**	45	32	12	1	44 (97.78%)
**F**					11.15
**P value**					<0.0001

### 3.2 Comparison of HAMD-17 scores and CGI-I scores before and after treatment between groups

Symptomatic differences were observed at baseline, and two and four weeks after treatment with rTMS in combination with fluoxetine in Sham, Low, and High rTMS groups. Initially, no significant differences in HAMD-17 scores were found between the groups (F = 0.071, P = 0.9316). However, after two and four weeks of treatment, both Low and High rTMS groups exhibited a more significant reduction from baseline compared to the Sham rTMS group (Low: 37.27 ± 7.16 vs. 16.22 ± 3.77, F =113.4, P <0.0001; High: 36.80 ± 5.51 vs. 15.35 ± 6.23, F =159.5, P <0.0001; Sham: 36.76 ± 7.79 vs. 21.31 ± 3.27; F = 64.9, P <0.0001). Specifically, Hamilton-17 depression scores in the Low rTMS group significantly decreased four weeks after treatment compared to the Sham rTMS group (16.22 ± 3.77 vs. 21.31 ± 3.27), while the High rTMS group, scores significantly decreased two weeks after treatment compared to the Sham rTMS group (25.84 ± 5.38 vs. 31.38 ± 7.51), as indicated in **Table 3**.

**Table 3:** Comparison of HAMD-17 scores before and after treatment between groups (n=135, $\bar{x}$ ± s, score).

Group	Case	Hamilton Depression Scale-17 (HAMD-17)			F	P value
		Baseline	Treatment for two weeks	Treatment for four weeks		
**Sham rTMS group**	45	36.76 ± 7.79	31.38 ± 7.51	21.31 ± 3.27*	64.9	<0.0001
**Low rTMS group**	45	37.27 ± 7.16	28.09 ± 6.87*	16.22 ± 3.77*#	113.4	<0.0001
**High rTMS group**	45	36.80 ± 5.51	25.84 ± 5.38*#	15.35 ± 6.23*#	159.5	<0.0001
**F**		0.071	7.889	15.900		
**P value**		0.9316	0.0006	<0.0001		

Statistical significance at p<0.05.

*p<0.001, compared with the Baseline level.

^#^p<0.001, compared with Sham rTMS level.

**Table 4** shows the comparisons of changes in CGI-I score. There were no statistically significant differences in CGI-I scores between the three groups in the baseline level (F = 0.072, P = 0.9308), but the differences between groups after two or four weeks of treatment are significant (two weeks: F =5.385, P = 0.0056; four weeks: F = 33.500, P<0.0001). Also in each group, the differences compared to the baseline were significant.

**Table 4:** Comparison of CGI-I scores before and after treatment between groups (n=135, $\bar{x}$ ± s, score).

Group	Case	CGI-I			F	P value
		Baseline	Treatment for two weeks	Treatment for four weeks		
**Sham rTMS group**	45	5.04 ± 0.47	3.36 ± 0.48*	2.47 ± 0.50*	324.1	<0.0001
**Low rTMS group**	45	5.00 ± 0.43	2.98 ± 0.66*	1.56 ± 0.50*#	467.2	<0.0001
**High rTMS group**	45	5.02 ± 0.72	2.89 ± 0.93*	1.42 ± 0.89*#	201.9	<0.0001
**F**		0.072	5.385	33.500		
**P value**		0.9308	0.0056	<0.0001		

Statistical significance at p<0.05.

*p<0.001, compared with the Baseline level.

^#^p<0.001, compared with Sham rTMS level.

### 3.3 Comparison of cognitive function scores between groups

The Low and Up rTMS groups exhibited a significant increase in the correct response count on the WCST compared to Baseline scores (P < 0.05). Likewise, the number of persistent and random errors on the WCST significantly decreased (P < 0.05), and the number of completed categories notably increased (P < 0.05). Furthermore, the High and Low rTMS groups showed superior cognitive function outcomes compared to the Sham rTMS group (P < 0.05), as shown in **Table 5**.

**Table 5:** Comparison of WCST scores before and after treatment between groups (n=135, $\bar{x}$ ± s, score).

Group	Times	Overall test	Correct response	Sustained fault	Random error	Complete classification
**Sham rTMS group**	**Baseline**	91.42±6.73	60.82±2.96	26.36±2.64	39.62±1.92	2.09±0.79
	**Post**	99.09±10.51^*^	69.60±4.04^*^	14.71±2.02^*^	23.96±1.98^*^	2.98±0.87^*^
**Low rTMS group**	**Baseline**	90.00±5.09	59.67±2.58	25.13±2.23	39.47±2.70	2.02±0.87
	**Post**	117.50±11.09^*#^	80.24±5.27^*#^	9.82±2.04^*#^	16.27±2.91^*#^	3.98±1.14^*#^
**High rTMS group**	**Baseline**	00.04±5.66	57.04±5.35	25.42±3.78	37.64±4.88	2.00±0.67
	**Post**	119.04±11.50^*#^	81.27±14.06^*#^	8.87±5.35^*#^	16.33±4.86^*#^	4.46±0.99^*#^

Statistical significance at p<0.05.

*p<0.001, compared with the Baseline level.

^#^p<0.001, compared with Sham rTMS level.

### 3.4 Comparison of adverse reactions between groups

Neither group experienced severe adverse events such as seizures or syncope. Minor adverse reactions observed included transient nausea and dizziness. There was no statistically significant difference in the incidence of adverse reactions between the two groups (F = 4.421, P = 0.0794), as shown in **Table 6**.

**Table 6:** Comparison of adverse reactions between groups [n=135, n (%)].

Group	Cases	Nausea	Dizziness	Elevation of blood pressure	Drowsiness	Total incidence(%)
**Sham rTMS group**	45	1	1	0	4	6 (13.33%)
**Observation group**	45	0	1	1	5	7 (15.56%)
**Low rTMS group**	45	2	3	2	4	11 (24.4%)
**F**						4.421
**P value**						0.0794

## 4 Discussion

In recent years, the incidence of depression has increasingly impacted younger populations, with the detection rate of adolescent depression rising annually (24). Patients commonly present somatic symptoms such as insomnia, headaches, fatigue, loss of appetite, and limb pain, alongside psychological symptoms like low mood, sluggish reactions, and abnormal thinking (28, 29). Without effective treatment, the persistent negative emotions experienced by adolescents can disrupt their normal learning and daily activities, causing significant detriment to their physical and mental health. Pharmacotherapy, primarily involving fluoxetine, increases brain serotonin levels, providing antidepressant effects and enhancing cognitive functions (30). However, clinical studies indicate that the long-term outcomes of relying solely on antidepressant medications are suboptimal, with frequent recurrences and notable side effects (30). Consequently, non-pharmacological interventions are increasingly used alongside pharmacotherapies.

### 4.1 Analysis of the overall effectiveness of combining rTMS and fluoxetine versus fluoxetine alone in treating adolescent depression

rTMS is a noninvasive neuromodulatory technique that utilizes electromagnetic induction to target specific brain areas. This process not only boosts brain metabolism and cortical excitability but also augments the effectiveness of antidepressants, leading to a rapid alleviation of clinical symptoms (31–33). Studies have demonstrated that rTMS can reduce depressive symptoms by restoring insular functional connections. In this study, 135 patients were administered either fluoxetine alone or a combination of fluoxetine and rTMS. The results showed an overall effective rate of 95.56% and 97.78% for the combined treatments, markedly higher than that for fluoxetine alone (80.0%), indicating that rTMS amplifies drug efficacy and improves clinical outcomes in first-episode adolescent depression. The mechanism involves stimulating the left dorsolateral prefrontal lobe, affecting the limbic system-thalamic-cortical neural network, increases blood perfusion, and regulates neuroendocrine-related factors (31–33). These changes enhance synaptic and neuronal plasticity, boosting excitability and cognitive function.

### 4.2 Using HAMD-17 and CGI-I scores to analyze the efficacy of combining rTMS with fluoxetine versus fluoxetine alone in treating adolescent depression

This study aimed to assess the impact of combining rTMS with fluoxetine on improving symptoms of adolescent depression. The HAMD-17 is an effective tool for measuring depression severity (34), while the CGI-I evaluates therapeutic effect (35). Quantitative analysis using these measures indicated that rTMS combined with fluoxetine was significantly more effective than fluoxetine alone in ameliorating symptoms of adolescent depression. Additionally, there was no statistical difference in the incidence of adverse reactions between the treatment groups, affirming that fluoxetine combined with rTMS can enhance efficacy while maintaining safety. Consistent with previous studies (36), rTMS used with fluoxetine significantly improves clinical outcomes and effectively relieves depression without impairing cognitive function, showing potential for specific enhancements. It is hypothesized that rTMS increases dopamine neurotransmitter secretions in the hippocampus and striatum. In conjunction with fluoxetine, these treatments mutually reinforce each other, elevating the patientng mood and cognitive functions (36). Furthermore, during rTMS, high-frequency magnetic stimulation applied to the left prefrontal lobe and low-frequency stimulation to the right modulate the cerebral cortex, subcortical pathways, and limbic system, balancing excitation and inhibition to improve emotional and cognitive functions. Ultimately, the treatment is deemed safe, with no severe adverse reactions, and does not impede the therapeutic process, aiming to improve the patient’s quality of life and prognosis (12, 32).

### 4.3 Combined treatment of rTMS and fluoxetine promotes greater improvement in cognitive functions of depression patients compared to fluoxetine alone

Cognitive impairment, including language deficits, memory loss, and attention difficulties, is a central symptom of depression that persists throughout the illness and significantly impairs social functioning (29, 30, 37). This study showed that after treatment, the observation group exhibited a significant reduction in false responses and persistent errors on the WCST (26), along with a notable increase in completed classifications compared to the Sham rTMS group. These results suggest that the combination of fluoxetine and rTMS markedly improves cognitive function in adolescents experiencing their first depressive episode. The improvement is attributed to rTMS boosting cerebral cortex metabolism, increasing cerebral blood flow, and regulating neural signal transduction, all of which collectively enhance perception and memory abilities (31, 32, 38). Concurrently, fluoxetine alters neurotransmitter expression and enhances hippocampal neural function (10, 39). Together, these treatments significantly improve cerebral circulation, thereby boosting cognitive functions (40).

### 4.4 Future challenges and prospects

Transcranial magnetic stimulation, recognized as a neuromodulatory technique with high efficacy, safety, and minimal adverse reactions, offers broad application prospects in clinical practice. However, challenges remain in treating adolescent depression due to non-specific parameter settings and unclear brain and neurophysiological mechanisms. First, the treatment of rTMS in adolescents still utilizes adult parameters as the standard. Given that adolescents exhibit lower tolerance for adverse reactions to rTMS, it is necessary to establish standardized rTMS usage guidelines for this age group. Second, although rTMS is a high-safety neuromodulatory therapy technique with few serious adverse reactions, further studies are needed to optimize treatment parameters, such as reducing headache-inducing stimulation times. Third, rTMS combined with drugs or psychotherapy has been shown to improve depressive symptoms more effectively, but the efficacy and mechanisms of the combined therapy remain to be elucidated. Exploring a combination therapy with high feasibility and effectiveness is crucial for future research. Fourth, research indicates that rTMS therapy can regulate the pathological imbalance of gamma-aminobutyric acid (GABA) (40) and glutamate (41). Going forward, it will be vital to explore the neurophysiological mechanisms and brain mechanisms in adolescents to better tailor treatment plans.

### 4.5 Conclusions

In conclusion, the combined use of rTMS and fluoxetine in treating first-episode adolescent depression not only alleviates depressive symptoms more effectively but also significantly enhances cognitive function. This approach demonstrates substantial efficacy and high safety, offering valuable insights for the clinical treatment of early-onset depression in adolescents.

## Data availability statement

The raw data supporting the conclusions of this article will be made available by the authors, without undue reservation.

## Ethics statement

Written informed consent was obtained from the minor(s)’ legal guardian/next of kin for the publication of any potentially identifiable images or data included in this article.

## Author contributions

LJ: Conceptualization, Data curation, Formal analysis, Investigation, Project administration, Writing – original draft. TC: Formal analysis, Funding acquisition, Resources, Software, Writing – original draft. YH: Data curation, Methodology, Resources, Writing – review & editing. XH: Supervision, Validation, Writing – review & editing.
